# On the Secretion of Sugar in the Animal Economy

**Published:** 1856-04

**Authors:** Bernard Henry


### ON THE SECRETION OF THE SUGAR IN THE ANIMAL ECONOMY.

BY BERNARD HENRY, M. D.

Dr. Henry, in a late number of the Medical Examiner, has prepared a very accurate and interesting *resume* of the investigations of the French and German physiologists with regard to the secretion of sugar in the human body. He alludes to the experiments of Lehman, Bernard, and Andral on this subject, and calls attention to the attack of M. Figuer upon Bernard's theories, which we have already noticed in our journal, (Vol. V. No. IV). Dr. Henry considers that we may correctly arrive at the following conclusions:

That sugar is a normal product in man.

That this principle is secreted in the liver, and that it is a normal function of that organ.

That the source of its supply is from nitrogenized elements.

That the food furnishes it also to the system.

That in the glycogenic function there is a sympathy of relation between the liver, the lungs and the cerebral center.

That in the disease called diabetes mellitus, the equilibrium of the production and destruction is disturbed, and that any one of these three structures may be at fault; and that it is to one or more of them that our remedies must be directed.

That the experiments of Lehman, Bernard, and Andral will warrant the careful allowance of small portions of vegetable food in this disease, and thus relieve our patients from one of the most distressing and trying attendants of the present mode of treatment.

That the labors of the physiologists, and above all, of Mr. Claude Bernard, have paved the way for a better understanding of diabetes mellitus, by demonstrating the condition of the glycogenic function in the state of health; but that close and more extended pathological observations are called for to render his researches available to the physician for a successful plan of treatment of a disease which is rare, but has thus far proved intractable.—*Virginia Medical Journal.*

### THE NATURE AND TREATMENT OF ENCEPHALO-MENINGITIS.

Dr. Leon Liegard, whose special attention has been drawn to this disease by the excessive fatality of meningitis of children in the Hopital de l'Enfant Jesus, and by the apparent inefficacy of the various remedies of an opposite character administered, supports M. Robin's opinion, that the granulations met with in these cases are not of a tuberculous character, and that we must therefore materially modify our views with regard to the nature and treatment of the disease. It appears that the medical officers of the hospital had been so discouraged by the inutility of applying either the antiphlogistic, the alterative, or the tonic mode of treatment, that they abandoned therapeutic attempts altogether; and, a diagnosis of meningitis having been established, resigned their patients to their fate. The views advocated by the author are mainly based upon Robin's micrographic account of the granulations, so that it is necessary to give a summary of his description.

Robin assumes two varieties. The first is of a yellowish tint, softish to the touch, and friable. Nine-tenths of these granulations consist of a finely granular amorphous matter; they contain a small quantity of cytoblasts, a few capillaries, and occasionally fibro-plastic formations. The second may, and most frequently does, co-exist with the former in the meninges, and is also found co-incidently in other serous membranes. It is the form specially known under the term gray or semi-transparent granulation. Robin finds that the bulk of the product is composed of the same amorphous granular matter as that above described, but that it is firmer; the cytoblasts are in large numbers, as is particularly manifest on the addition of acetic acid. Fibro-plastic elements, and a network of fine areolar tissue, accompanied by scanty vessels, also present. These cytoblasts are described as diffeiing from the tubercle corpuscles (1) in being polyhedric, with irregular and slightly-toothed margins, instead of oval or round; (2) their diameter averages half the size of the latter; (3) the tubercle corpuscle is rendered pale by acetic acid, but is not dissolved; the contour of the cytoblasts is rendered more defined; (4) the granulations contained in the tubercle corpuscle are scattered uniformly through the corpuscle, while in the cytoblasts they are congregated in the middle; (5) although the cytoblasts contain no nucleolus, they belong to the cellular element which commonly exhibits the presence of free nuclei. We scarcely ever find the variety of free nuclei without meeting with some cells containing a nucleus resembling the free nuclei.

Assuming this definition of M. Robin's to be correct, the author regards the inflammation which determines the formation of the granulations to be of a specific character, analagous to that of croup, and attributes the fatality of the disease in the children's hospital to the erroneous assumption that it was of a tuberculous character. M. Liegard then quotes several cases diagnosed as tubercular or granular meningitis, in which cures were obtained by powerful antiphlogistic remedies, especially by venesection or leeches to the mastoid processes, and mercurial inunctions over the abdomen, repeated every three hours, with calomel administered internally. The author admits the impossibility of diagnosing the granular from the simple form of meningitis, and proposes the classification of the disease, according to the violence of the symptoms, and the rapidity of its course, into the inflammatory and sub-inflammatory forms. According to the intensity of symptoms, he recommends a variation of the treatment, not in kind, but in degree; and he concludes, both from the character of the morbid changes and the results obtained on the basis of the views advocated, that this fearful malady may be successfully combated by the means proposed, and ought not to be regarded as necessarily fatal.—*B. & F. Med. Chirurg. Rev.*, Oct. 1855, from *Rev. Medico-Chirurg. de Paris*, Jan. and Feb. 1855.

